# Commentary: COVID-19 impairs oxygen delivery by altering red blood cell hematological, hemorheological, and oxygen transport properties

**DOI:** 10.3389/fphys.2024.1426505

**Published:** 2024-09-10

**Authors:** Dieter Böning, Wilhelm Bloch, Dominik Vogel, Mathias Steinach, Wolfgang M. Kuebler

**Affiliations:** ^1^ Institute of Physiology, Charité ‐ Universitätsmedizin Berlin, Berlin, Germany; ^2^ Department of Molecular and Cellular Sport Medicine, Institute of Cardiovascular Research and Sport Medicine, German Sport University Cologne, Cologne, Germany; ^3^ Medical Chamber for Lower Austria, Vienna, Austria; ^4^ Center for Space Medicine and Extreme Environments Berlin, Charité - Universitätsmedizin, Berlin, Berlin, Germany

**Keywords:** hemoglobin oxygen affinity, *in vivo* oxygen dissociation curve, hemox-analyzer, 2,3-bisphosphoglycerate, erythrocytes

## Introduction

Recently, Rogers and colleagues (Rogers et al., 2023) published an article on the effects of COVID-19 on “Oxygen Delivery by Altering Red Blood Cell Hematological, Hemorheological, and Oxygen Transport Properties“ in this journal. Venous samples from hospitalized patients were compared to blood of healthy controls.

One part of their study was the measurement of oxygen affinity, characterized by the half saturation pressure (P_50_) and the slope n in the logarithmic Hill plot at standard conditions (pH 7.2, 7.4, 7.6, 37°C). These measurement revealed a tendency for slightly increased P_50_ and decreased n values in 11 COVID-19 patients relative to 14 controls at all pH values, yet apparently without reaching the level of significance (P_50_ at pH 7.2: 28.77 ± 1.87 vs. 29.83 ± 2.31 mmHg, *p* = 0.2198; pH 7.4: 23.06 ± 1.69 vs. 24.3 ± 2.25 mmHg, *p* = 0.124; pH 7.6: 18.69 ± 1.67 vs. 19.59 ± 1.72 mmHg, *p* = 0.1929, healthy control vs COVID-19, respectively). The authors therefore concluded that COVID-19 exerts no effect on hemoglobin oxygen affinity.

## Comments

We have some concerns with this interpretation, which can be summarized as follows:

Statistics: First, the statistical analysis only applied individual t-tests for each pH value, rather than analyzing variance for the complete data set. Our analysis (data supplied by Rogers et al., method “GraphPad Prism 9.3.1. (built 471) for Windows 64 bit, GraphPad Software, San Diego, California, USA”) revealed a significant group-effect between healthy subjects and COVID-19 patients (*p* = 0.0114) leading to greater values of standard-P_50_ at all investigated pH-values among COVID-19 patients (See Table 1).

**TABLE 1:** Statistical analysis performed with the whole data set supplied by Rogers et al. through Grouped Two-way ANOVA.

Grouped: Two-way ANOVA			
Source of variation	% of total variation	f-value	*p*-value
Interaction	0.07225	0.1568	0.8552
Row factor (pH-values)	81.40	176.6	<0.0001
Column Factor (Healthy vs. Covid-19 patients)	1.558	6.762	0.0114

As venous blood was investigated, this corresponds to the right shift in such samples described in our recent review in this journal (Böning et al., 2023) supporting oxygen delivery to tissue. The corresponding small decrease of Hill`s n is negligible.

Method: Second, the authors used the Hemox-Analyzer (Asakura, 1979; Guarnone et al., 1995) for registration of oxygen dissociation curves (ODC). In this apparatus venous blood was extremely diluted (120-fold) and analyzed under artificial conditions (no CO_2_, diluted in 3 mL of 50 mM BIS-Tris, and 100 mM NaCl, buffered to either pH 7.2, 7.4 or 7.6). Important extrinsic effects by substances like CO_2_, bicarbonate, or lactic acid directly affecting hemoglobin oxygen affinity, or ions that affect membrane potential like potassium or calcium and thus change intraerythrocytic pH are lacking in this assay, or are only present in subphysiological concentrations which will inherently affect the ODC. Consistently, the results correspond in part to former publications applying the same methodology who also failed to detect marked changes of the ODC in COVID-19 (Daniel et al., 2020; Renoux et al., 2021). Notably, however, these studies reported higher standard P_50_ values in both patients and healthy control subjects (>27 mmHg).

Comparison to publications applying different methods: Third, the reported effects of COVID-19 on the OCD were, however, variable in other investigations with different methodological approaches. Most investigators calculated P_50_ from single measurements in native blood with blood gas analyzers applying correction factors for pH and PCO_2_. In several articles a shift of the ODC (mainly leftward in arterial and rightward in venous blood) was observed. We have reviewed these studies last year in a publication in this journal (Böning et al., 2023); they are, however, not considered, cited or discussed in the recent work by Rogers and colleagues. The authors also fail to explain the rather low standard P_50_ values of, e.g., 23.1 mmHg for the control group at pH 7.4. Generally, P_50_ values amount to approximately 26–28 mmHg, with slightly higher values for females before menopause than for males (Humpeler et al., 1989).

2,3-Biphosphoglycerate: Fourth, as in virtually all studies on the ODC in COVID-19 so far, the concentration of 2,3-bisphosphoglycerate (2,3-BPG) as a critical determinant of the P_50_ was not measured. Until recently only Thomas and colleagues (Thomas et al., 2020) had communicated slighty elevated 2,3-BPG concentrations in moderately ill COVID-19 patients, but only arbitrary units were used. The reason for the relative lack of reported 2,3-BPG levels seems to be that most major companies (e.g., Roche, Sigma) had ended the production of the kits for photometric measurements prior to the pandemic. Yet, a new study on 2,3-BPG concentration was recently published as preproof by Bertilacchi and coworkers (Bertilacchi et al., 2024). Here, the authors applied a competitive enzyme-linked immunosorbent assay (ELISA, COD. MBS288269-96, MyBioSource) to measure 2,3-BPG levels in sixty eight patients on the intensive care unit. Patients were stratified into two subgroups based on a median arterial oxygen pressure of 67 mmHg. Interestingly, the mean value for 2,3-BPG (corrected by us for a calculation error) was 4.9 ± 1.4 mmol/L erythrocytes for patients with PaO_2_ > 67 mmHg, but 5.7 ± 1.5 mmol/L for patients with PaO_2_ < 67 mmHg. Both values are higher than in healthy subjects at sea level (approximately 4 mmol/L according to (Duhm and Gerlach, 1974)), and are consistent with the typical response to hypoxia (reviewed in West et al., 2007).

## Discussion

Based on these considerations, we suggest that the measurement of P_50_ with the Hemox-Analyzer in the study by Rogers and colleagues likely yields lower than actual half saturation pressures, and potential effects of COVID-19 such as a moderate decrease in oxygen affinity as formerly observed in venous blood failed to reach significance based on the applied statistical evaluation. Direct measurements of 2,3-BPG by Bertilacchi and colleagues (Bertilacchi et al., 2024) suggest increased concentrations in COVID-19 patients. Unfortunately these authors did not calculate half saturation pressures, or report 2,3-BPG values in healthy controls. As such, the ultimate word on the effects of COVID-19 on ODC, and the potential role of altered 2,3-BPG levels in this scenario still await to be elucidated.
